# CRRT influences PICCO measurements in febrile critically ill patients

**DOI:** 10.1515/med-2022-0430

**Published:** 2022-02-14

**Authors:** Qiancheng Xu, Yuhan Cao, Weihua Lu, Jianguo Li

**Affiliations:** Department of Critical Care Medicine, Zhongnan Hospital of Wuhan University, Wuhan 430071, Hubei, China; Department of Critical Care Medicine, The First Affiliated Hospital of Wannan Medical College (Yijishan Hospital of Wannan Medical College), Wuhu, 241000, Anhui, China; Department of Nephrology, The First Affiliated Hospital of Wannan Medical College (Yijishan Hospital of Wannan Medical College), Wuhu, 241000, Anhui, China

**Keywords:** continuous renal replacement therapy, global end-diastolic volume index, cardiac index, pulse index continuous cardiac output, hemodialysis, fever

## Abstract

The aim of this study was to investigate whether continuous renal replacement therapy (CRRT) influences the global end-diastolic volume index (GEDVI), cardiac index (CI), and extravascular lung water index (EVLWI) measured by Pulse Index Continuous Cardiac Output (PICCO) in febrile patients. Fifteen fever patients were included in this study. CI, GEDVI, EVLWI, heart rate (HR), and mean arterial pressure (MAP) were measured at five time-points: before CRRT (T0), immediately after CRRT started (T1), 15 min after CRRT started (T2), immediately after CRRT stopped (T3), and 15 min after CRRT stopped (T4). Results have shown that CI and GEDVI were decreased significantly in T1 (CI: 4.09 ± 0.72 vs 2.81 ± 0.58 L/min m^2^, *P* = 0.000 and GEDVI: 727.86 ± 63.47 vs 531.07 ± 66.63 mL/m^2^, *P* = 0.000). However, CI and GEDVI were significantly increased in T3 (CI: 4.09 ± 0.72 vs 7.23 ± 1.32 L/min m^2^, *P* = 0.000 and GEDVI 727.86 ± 63.47 vs 1339.17 ± 121.52 mL/m^2^, *P* = 0.000). There were no significant differences in T2 and T4. Among the five-time points, no measurement errors were observed with regards to HR, MAP, and EVLWI. Therefore, the data herein contained suggests that PICCO measurements should begin 15 min after the start or stop of CRRT.

## 1 Introduction

Hemodynamic instability is one of the most common syndromes present in critically ill patients and is associated with high mortality rates [1,2,3]. These patients require hemodynamic monitoring to guide vasopressor usage and gauge the need of volume resuscitation [4]. Pulse Index Continuous Cardiac Output (PICCO) is one of the most effective methods to provide accurate cardiovascular parameters, such as the volume index, cardiac function, peripheral vascular resistance, and stroke volume variation [5,6]. Critically ill patients with acute kidney injury (AKI) are common in the intensive care unit (ICU), with an AKI incidence varying from 15–81% [7,8,9]. Continuous Renal Replacement Therapy (CRRT) is widely used to treat AKI patients due to accurate volume control, steady acid-base and electrolyte corrections, and stabilization of hemodynamic parameters [10,11,12].

PICCO measurements are based on thermodilution [5]; however, CRRT can influence blood temperature and distribution. Therefore, PICCO parameters are greatly affected immediately after starting and stopping CRRT [13]. This influence disappears after a few minutes of starting or stopping the CRRT [14]. However, patients with fever due to systemic inflammatory response syndrome, secondary to sepsis or surgery, are prevalent in ICUs [15,16]. When fever patients are subjected to CRRT, the clinician usually sets the temperature of the hemofiltration replacement fluid in a “lower range” to decrease the odds of developing hyperpyrexia, which may cause great disturbances in blood temperature, thus leading to more measurement errors than those of non-febrile patients, even after CRRT is stable. Therefore, this study aimed to determine whether CRRT influences PICCO measurements in fever patients.

## 2 Patients and methods

### 2.1 Study population

This prospective observational study was conducted in a general ICU of a university hospital. Fifteen fever patients were enrolled in this study. All patients were monitored by PICCO and treated with CRRT from July 2018 to July 2019. The clinical indications for CRRT were severe hypervolemia, severe hyperkalemia, severe heart failure with no response to diuretics, uremic encephalopathy, and other reasons determined by the attending physicians in the ICU or the nephrology department [17]. The indication for PICCO was hemodynamic instability that could not be corrected by fluid resuscitation in a short period by using other methods such as CVP, volume challenge, passive leg raising (PLR) test, or echocardiography [18]. Protocols involving patients were complied with all the relevant national regulations, institutional policies, and in accordance with the Helsinki Declaration, and were approved by the Institutional Review Board of the First Affiliated Hospital of Wannan Medical College (2016–2019). Written informed consent was obtained from each patient or his/her authorized representatives.

### 2.2 PICCO measurements

The PICCO plus equipment used in this study was produced in Germany (PulsioCath, PV2015L20 N, Pulsion Medical Systems, Munich, Germany). The internal jugular or subclavian veins were catheterized by a double-lumen catheter (8.5 Fr, 16 cm in length CS-22854-E, Arrow, Everett, Ma), which was used to inject cold boluses and perform PICCO. X-ray was used to confirm that the catheter tip was positioned within the superior vena cava before injecting cold saline. The femoral artery was catheterized by a 5-Fr thermistor-tipped catheter (PulsioCath, PV2015L20 N, Pulsion Medical Systems, Munich, Germany). Cold saline injections (<8°C) were injected three times for measurements. Average measurements recorded consisted of global end-diastolic volume index (GEDVI), cardiac index (CI), extravascular lung water index (EVLWI), Heart Rate (HR), and mean arterial pressure (MAP). Patients with significant fluctuations in measurements or vital signs (variation greater than 10%) were excluded [6,19].

### 2.3 CRRT

The CRRT device (Prismaflex System) used in this study was produced by Gambro. The dialysis catheter was injected into the femoral vein (14 Fr, 25 cm length, two lumens, CS-26142-F, Arrow, Everett, Ma.) that was contralateral to the arterial catheter. Circuit components, including the hemofilter (Prismaflex M100 set, Gambro Industries, France), were also produced by Gambro. The CRRT parameters were set as follows: continuous venou venous hemofiltration model, blood flow of 100 mL/h for all patients, the predilution flow rate of 2,000 mL/h, and postdilution flow rate of 1,000 mL/h, with no fluid loss during the examination period, and temperature set at 37°C. Heparin was pumped continuously at 5–20 units/kg/h for anticoagulation and to maintain activated clotting time (ACT) at 180–220 s. ACT was monitored every 4 h [17]. If a patient was considered to have a high risk of bleeding, no anticoagulant was used.

### 2.4 Measurement protocol (Figure 1)

**Figure 1:**
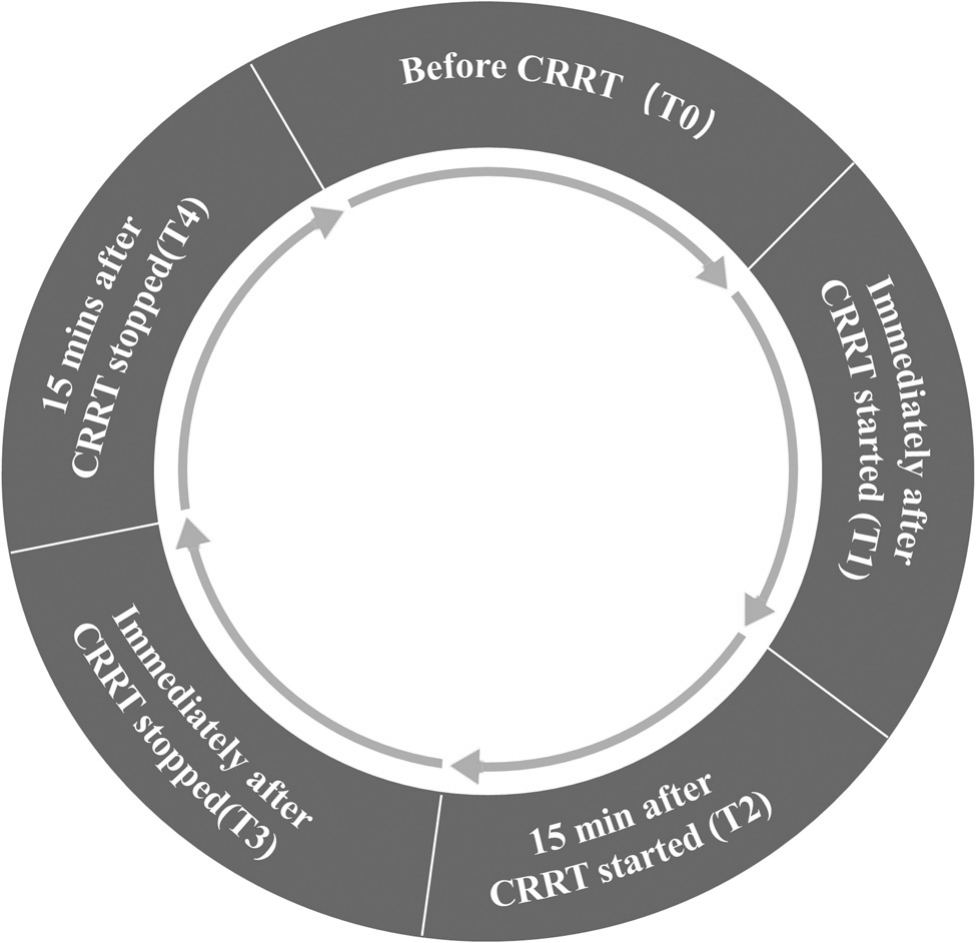
Study protocol and PICCO measurements at different time points. CRRT: continual renal replacement therapy; T0: before CRRT; T1: immediately after CRRT started; T2: 15 min after CRRT started; T3: immediately after CRRT stopped; T4: 15 min after CRRT stopped.

The CI, GEDVI, EVLWI, HR, MAP, and body temperature were measured by PICCO under the following time-points: before CRRT (T0), immediately after CRRT started (T1), 15 min after CRRT started (T2), immediately after CRRT stopped (T3), and 15 min after CRRT stopped (T4). Treatments and CRRT parameters were kept the same during measurements. Two measurements were taken for each patient with an interval of 24 h.

### 2.5 Statistical analysis

Continuous variables were summarized either as mean value and standard deviation (mean value ± SD) or median and interquartile range (IQR). Categorical variables were described as frequencies and percentages. Differences in hemodynamic data were analyzed using Fisher’s exact test or the Wilcoxon signed-rank test for categorical variables, and paired student *t*-test for continuous variables. A *p* < 0.05 was considered statistically significant. All analyses were performed using the SPSS 15.0 software.


**Ethics approval consent to participate:** This study was approved by The First Affiliated Hospital of Wannan Medical College (Yijishan Hospital of Wannan Medical College) Medical Ethics Committee (No. 2016-19). Written consent was obtained from each patient or his/her authorized representatives.

## 3 Results

Fifteen patients were included in this study. Measurements were taken two times in all patients, with an interval of 24 h and one set of data was excluded due to significant changes in vital signs during measurement. Therefore, 29 sets of data were recorded for 15 patients.

The baseline characteristics of the population are summarized in Table 1. The median patient age was 70.9 years and 60% (9/15) patients were male, while the mean acute physiology and chronic health evaluation II (APACHE II) score was 21.3. Septic shock (33.3%, 5/15) and cardiogenic shock (66.7%, 10/15) were the main indications for PICCO monitoring. All patients (15/15) received mechanical ventilation, 86.7% (13/15) were treated with catecholamines. Hypertension and coronary heart disease were the main comorbidities. The average temperature was 38.87°C.

**Table 1:** Demographics, laboratory tests, vascular access, and ventilator parameters of included patients (*n* = 15)

Variable	Result
Age (years)	70.93 ± 13.42
Male (%)	9 (60%)
Weight (kg)	59.94 ± 9.63
Body height (cm)	166.34 ± 10.57
Vital sign	
Body temperature (°C)	38.87 ± 0.40
HR (bpm)	113.82 ± 18.86
Respiratory rate (bpm)	16.53 ± 1.52
MAP (mmHg)	68.17 ± 13.26
Patients with mechanical ventilation (*n*, %)	15 (100%)
FiO_2_ (%)	55.0 (40.0–60.0)
PEEP (cmH_2_O)	8.0 (5.5–11.0)
Patients receiving catecholamines (*n*, %)	13 (86.67%)
Norepinephrine	9 (60%)
Epinephrine	4 (26.67%)
Dose of norepinephrine (ng/kg/min)	526.28 ± 120.91
Dose of epinephrine (ng/kg/min)	381.33 ± 121.87
Classification of shock (*n*, %)	
Septic shock	10 (66.67%)
Cardiogenic shock	5 (33.33%)
APACHE II score	21.32 ± 8.23
Comorbidity (*n*, %)	
Hypertension	8 (53.33%)
Coronary heart disease	6 (40.0%)
Chronic lung diseases	3 (20.0%)
Diabetes mellitus	3 (20.0%)
Others	2 (8.0%)
Laboratory tests	
White blood cell count (×10^9^/L)	12.54 (8.86–15.32)
Hemoglobin (g/L)	103.61 (89.78–127.07)
Platelet count (×10^9^/L)	115.72 (89.54–136.76)
d-dimer (mg/L)	2.42 (0.72–3.91)
Alanine aminotransferase (U/L)	89.83 (53.18–231.37)
Albumin (g/L)	33.18 (31.58–37.33)
Creatine (mmol/L)	124.88 (79.62–198.13)
Lactic acid (mmol/L)	3.18 (2.61–4.73)
C reactive protein (mg/dL)	108.52 (37.88–184.29)

Results are expressed as mean value ± SD, *n* (%), IQR. APACHE II: acute physiology and chronic health evaluation II; bpm: beats per minute.

**Figure 2:**
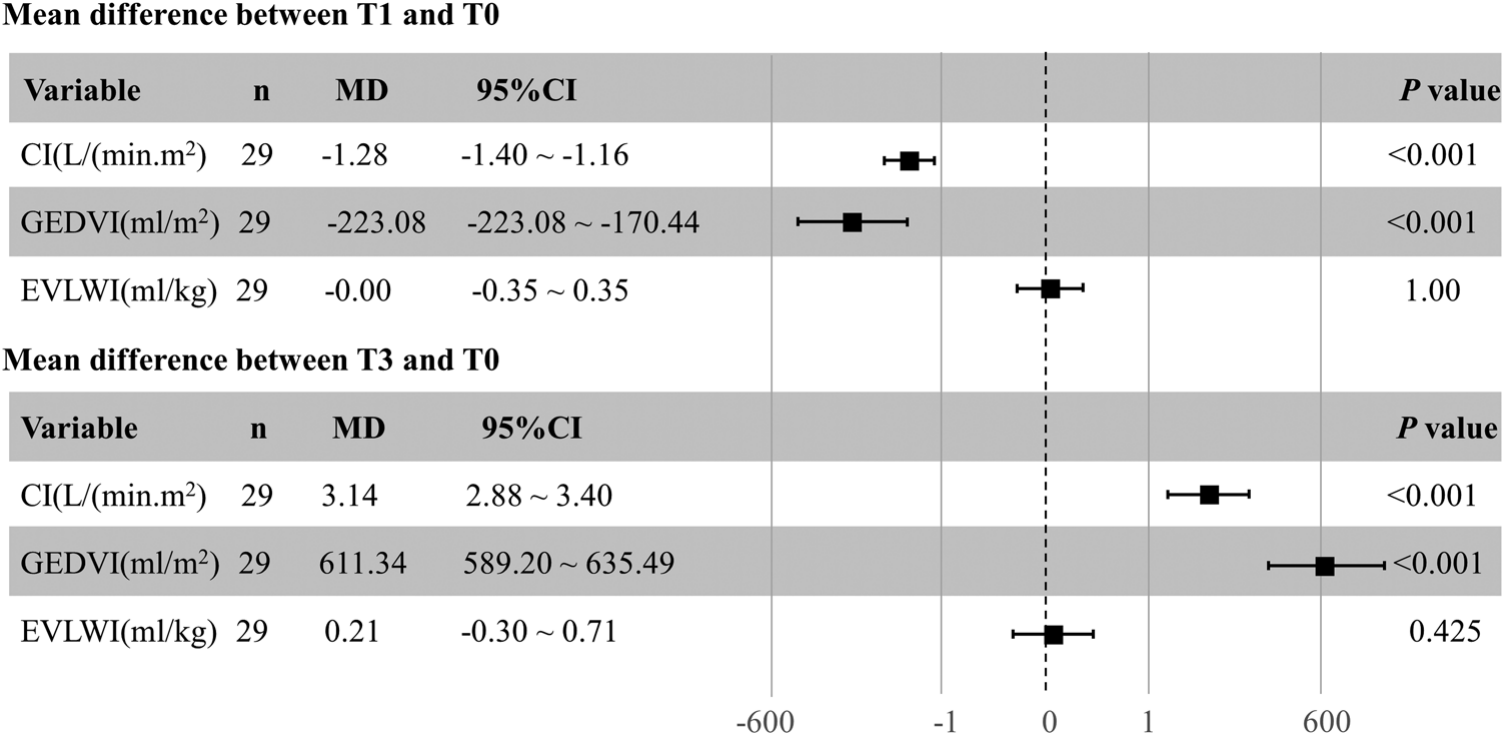
PICCO parameters changed at T1 and T3 when compared to baseline measurements (T0) from 29 datasets obtained from 15 patients. MD: mean difference; 95% CI: confidence interval; CI: cardiac index; GEDVI: global end-diastolic volume index; CRRT: continual renal replacement therapy; T0: before CRRT; T1: immediately after CRRT started; T3: immediately after CRRT stopped.

We found that compared to T0 during the CRRT treatment, fever patients monitored by PICCO presented significantly decreased CI and GEDVI during T1, (CI: 4.09 ± 0.72 vs 2.81 ± 0.58 L/min m^2^, *P* = 0.000 and GEDVI: 727.86 ± 63.47 vs 531.07 ± 66.63 mL/m^2^, *P* = 0.000). The mean change in CI was −1.28 (95% CI: −1.40 to −1.16 L/min m^2^, *P* = 0.00), and the mean change in GEDVI was −196.76 (95% CI: −223.08 to −170.44 mL/m^2^, *P* = 0.00). However, fever patients also presented increased CI and GEDVI in T3 compared to T0 (CI: 4.09 ± 0.72 vs 7.23 ± 1.32 L/min m^2^, *P* = 0.000 and GEDVI: 727.86 ± 63.47 vs 1339.17 ± 121.52 mL/m^2^, *P* = 0.000). The mean change in CI was +3.14 (95% CI: +2.88 to +3.40 L/min m^2^, *P* = 0.00), and the mean change in the GEDVI was +611.34 (95% CI: +589.20 to +635.49 mL/m^2^, *P* = 0.00). CI and GEDVI were similar during T2 and T4. No measurement error was observed in any time-point with regards to HR, MAP, and EVLWI (Table 2 and Figure 2).

**Table 2:** PICCO parameters (mean value ± standard deviation) changed by CRRT at different time points for 29 datasets obtained from 15 patients

Variable	T0	T1	T2	T3	T4
CI (L/min m^2^)	4.09 ± 0.72	2.81 ± 0.58*	4.09 ± 0.70	7.23 ± 1.32*	4.28 ± 0.77
GEDVI (mL/m^2^)	727.83 ± 63.47	531.07 ± 63.63*	735.48 ± 65.87	1,339.17 ± 121.52*	773.52 ± 71.06
EVLWI (mL/kg)	8.59 ± 2.75	8.59 ± 2.95	8.72 ± 2.93	8.79 ± 2.92	8.58 ± 2.75
HR (bpm)	97.66 ± 15.32	99.20 ± 16.24	97.76 ± 14.87	98.00 ± 15.24	101.21 ± 17.78
MAP (mmHg)	68.17 ± 13.26	68.49 ± 15.14	69.97 ± 13.73	70.41 ± 13.36	67.25 ± 14.76

Compared to T0, the asterisk indicates *P* < 0.01. CI: cardiac index; GEDVI: global end-diastolic volume index; EVLWI: extravascular lung water index; BT: body temperature; HR: heart rate; MAP: mean arterial pressure; CRRT: continual renal replacement therapy; T0: before CRRT; T1: immediately after CRRT started; T2: 15 min after CRRT started; T3: immediately after CRRT stopped; T4: 15 min after CRRT stopped.

## 4 Discussion

There were several clinically relevant findings in this study. First, CI and GEDVI were significantly influenced by CRRT in fever patients, immediately after the start and finish of CRRT. Indeed, both CI and GEDVI decreased significantly immediately after CRRT was started, while these parameters were significantly increased immediately after CRRT stopped. Second, 15 min after CRRT was started or stopped these changes were restored. Third, there were no measurement errors with regards to HR, MAP, and EVLWI.

It has been reported that PICCO measurements could be influenced by fluctuations in blood temperature [14,20]. We observed that the blood of patients could lose heat through the CRRT circuit because we have set the heater at a low range (35–37°C) to reduce the hyperpyrexia in patients. In this situation, blood returns to the body with a much lower temperature than the patient’s core temperature, leading to a continuous infusion of ‘cold’ liquid into the body while PICCO is being performed. Therefore, this may cause more pronounced measurement errors. Indeed, several studies have presented findings that corroborate our results. Heise et al. [14] demonstrated that the median core temperature of patients subjected to CRRT was 36.3°C (IQR 35.8–37.1°C), while the median temperature of the blood returning into the body from the CRRT circuit was 34.2°C (IQR 33.4–34.7°C). During disconnection of the CRRT circuit from the patient, the median blood temperature inside the device was significantly decreased to 31.6°C (median, IQR 30.6–31.2°C). Measurements performed after CRRT continuation has found that the median temperature of the returning blood was increased to 33.3°C (IQR 32.3–34°C). Compared with our study, we have only included patients with high fever, therefore the difference between the patient’s core temperature and the blood returning into the body from the CRRT circuit was greater than that reported by Heise et al. [14]. It can be inferred that measurement errors of CI and GEDVI were more pronounced in our study. Indeed, our measurement error is more pronounced than previous research by Heise et al. [14], suggesting a significant positive correlation between the temperature differences and measurement error.

According to the Stewat–Hamilton equation [21]:

{\text{CO}}_{\text{TDa}}\text{:}\hspace{.25em}({T}_{\text{b}}-{T}_{i})\cdot {V}_{i}\cdot K\text{/}\int \text{Δ}{T}_{\text{b}}\cdot \text{d}t.]

**Figure A1:**
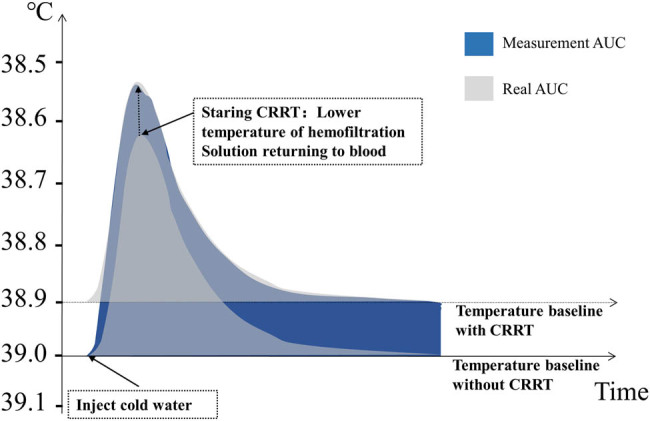
Changes of the AUC detected by PICCO at T1. The measured AUC was bigger than the real AUC.

**Figure A2:**
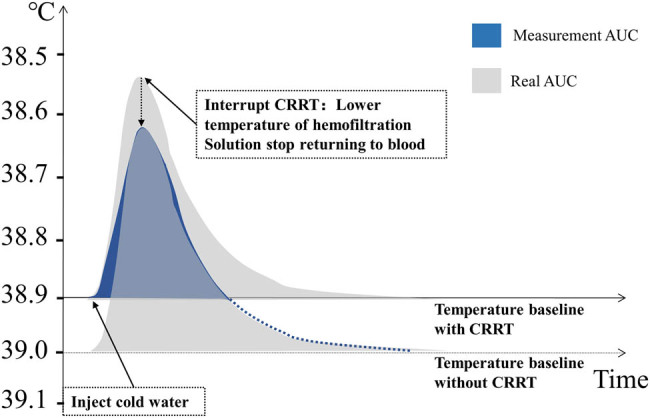
Changes of AUC detected by PICCO at T3. The measured AUC was smaller than the real AUC.

**Figure A3:**
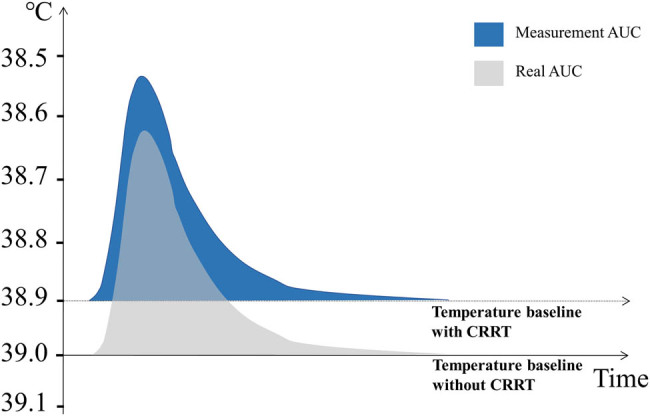
Changes of the AUC detected by PICCO at T2 and T4. The measured AUC was the same as the real AUC.

When the indicator dose and temperature are unchanged, the CI value is inversely proportional to the area under the curve (AUC) of the temperature dilution curve [21]. In our study, we found that CI and GEDVI decreased significantly immediately after CRRT began. This indicates that the lower temperature of the returning blood from the CRRT machine could increase the AUC and lead to miscalculation of both CI and GEDVI (Figure A1). Immediately after CRRT stopped, the abrupt halt of ‘cold’ blood returning to the body led to a decrease in the AUC, thus increasing CI and GEDVI (Figure A2). However, the temperature reaches a new steady-state after some time on CRRT, which results in a decreased baseline temperature that does not affect the AUC and, thus, does not lead to miscalculations of CI and GEDVI values (Figure A3), also verified in another study [22].

Previous studies have suggested that measurement errors may be caused by a “loss of indicator” due to modifications of the distribution of the cold bolus injected into the venous bloodstream [23,24,25]. The loss of an indicator in PICCO, such as a mispositioning of the central venous catheter tip, might have influenced CI measurements [11]. Martinez-Simon et al. [26] reported a case in which CRRT significantly decreased CI measurements. Authors have argued that this phenomenon occurred due to a non-laminar blood flow in the central vein caused by CRRT. However, we found that a three-lumen catheter was used in the report by Martinez-Simon, and the distance between orifices of a three-lumen catheter is minimal; therefore, the cold indicator could be injected through one lumen and suctioned through the other lumen, causing recirculation. This is also supported by the finding of a double peak in the temperature curves, which was supported by another study [14]. The position of the dialysis catheter tip (superior vena cava or inferior vena cava) also influences the cardiac output measurements of PICCO [27,28]. If the dialysis catheter and the catheter used to inject cold saline are in the same position, this could result in significant differences related to the ‘loss of the indicator’, as explained above. However, in our study, the two catheters were not positioned in the same area of the vena cava.

Previous research shows that a higher blood flow rate could result in the overestimation of both CI and GEDVI by PICCO. Sakka et al. [29] studied the influence of CRRT on CI by using transpulmonary thermodilution measurements. They have used a blood flow of 100 mL/min, and PICCO was performed after 15 min of CRRT. Changes in CI, intrathoracic blood volume index and EVLWI were, respectively, 0.1 L/min/m^2^, −18 mL/kg, and −0.07 mL/kg. Although the results were significantly different, they found no clinical significance. Therefore, in our study we have set the blood flow rate at 100 mL/min, and performed PICCO 15 min after CRRT was stopped, to avoid measurement errors. There is no definitive answer as to how much the blood flow rate influences CI measurements. Dufour et al. [30] found that a blood flow rate greater than 350 mL/h did not influence PICCO measurements during CRRT. However, this study performed PICCO measurements only after the CRRT run was stable. Indeed, we found that when the temperature reached a new steady state after the CRRT run was stable, we observed a decrease in the femoral artery baseline temperature that did not affect the AUC (Figure A3). However, we found significant measurement errors if the measurements were obtained immediately after CRRT started or paused. In our research, the lowest CI value was 2.3 L/min m^2^, which was the same as that found in the study by Sakka et al. [29]. Collectively, the position of the central venous catheter tip, and the blood flow rate had no influence on CI or GEDVI measurements in this study.

Our study has several limitations. First, because of the limitations of the experimental conditions in our study, we could not monitor the temperature of the blood returning into the body from the CRRT circuit. Furthermore, we did not investigate the relationship between low CI and high blood flow rate. These results would be interesting, and future experiments are planned to assess these. Finally, we cannot provide a reasonable explanation as to why the EVLWI was not influenced by CRRT.

In summary, CI and GEDVI measurements were significantly influenced in fever patients immediately after CRRT was started or stopped. However, there were no measurement errors 15 min after the CRRT run was stable or 15 min after it stopped. Therefore, it is recommended to start PICCO measurements only after CRRT has been running or stopped for more than 15 min.

## List of abbreviations

AKI: acute kidney injury
APACHE II: acute physiology and chronic health evaluation II
CI: cardiac index
CRRT: continuous renal replacement therapy
EVLWI: extravascular lung water index
GEDVI: global end-diastolic volume index
HR: heart rate
ICU: intensive care unit
IQR: interquartile ranges
MAP: mean arterial pressure
PICCO: pulse index continuous cardiac output
